# A 53-week, open-label phase IIIb study of velaglucerase alfa in Chinese patients with type 1 Gaucher disease: Safety, efficacy, and pharmacokinetics

**DOI:** 10.1016/j.ymgmr.2026.101324

**Published:** 2026-06-02

**Authors:** Lihui Zhang, Xue-Qun Luo, Yongjun Fang, Fengkui Zhang, Yan Meng, Xiaoping Luo, Liansheng Zhang, Zhengqing Qiu, Xiao Ge, Jaco Botha, Jenny Zhan, Tianyou Wang

**Affiliations:** a The Second Hospital of Hebei Medical University, Hebei, China; b The First Affiliated Hospital, Sun Yat-sen University, Guangdong, China; c Nanjing Children's Hospital, Nanjing, Jiangsu, China; d Institute of Hematology and Blood Diseases Hospital, Chinese Academy of Medical Sciences, Tianjin, China; e Chinese PLA General Hospital, Beijing, China; f Guangzhou Women and Children's Medical Center, Guangzhou Medical University, Guangzhou, China; g Tongji Medical College Hospital of Huazhong University of Science & Technology, Hubei, China; h Lanzhou University Second Hospital, Lanzhou, Gansu, China; i Peking Union Medical College Hospital, Beijing, China; j Takeda APAC Biopharmaceutical Research and Development Co., Ltd., Shanghai, China; k Takeda Pharmaceuticals International AG, Glattpark, Zurich, Switzerland; l Beijing Children's Hospital, Capital Medical University, Beijing, China

**Keywords:** China, Gaucher disease, velaglucerase alfa, pharmacokinetics

## Abstract

**Background:**

Velaglucerase alfa is approved in China for treating type 1 Gaucher disease (GD1), but data on its use in Chinese pediatric and adult patients are limited. This study evaluated the safety, efficacy, and pharmacokinetics of velaglucerase alfa in Chinese patients with GD1.

**Methods:**

This was a phase IIIb, multicenter, open-label, single-arm, 53-week study (NCT05529992). Patients received intravenous velaglucerase alfa (60 U/kg) every 2 weeks. The primary endpoint was the incidence of serious treatment-emergent adverse events (TEAEs). Secondary endpoints evaluated safety (incidence of TEAEs), efficacy (changes in hemoglobin concentrations, platelet counts, liver and spleen volumes [as percent of body weight, % BW]), and quality of life (QoL).

**Results:**

Twenty patients were enrolled (treatment-naïve, *n* = 16; previously treated, *n* = 4). Four patients experienced 4 serious TEAEs requiring hospitalization; none were treatment-related. Overall, 19 of 20 patients experienced 104 TEAEs, of which 9 were treatment-related (8.7%; all mild). No TEAEs or deaths led to discontinuation of treatment. Mean (SD) improvements in hemoglobin concentrations (baseline, 10.5 g/dL [2.2]; change, +2.3 g/dL [1.3]; +25.5%), platelet counts (baseline, 59.9 × 10^9^/L [23.4]; change, +42.1 × 10^9^/L [27.7]; +82.3%), normalized spleen (baseline, 5.2% BW [2.6]; change, −3.1% BW [1.6]; −58.4%), and liver volume (baseline, 4.6% BW [1.3]; change, −1.1% BW [0.9]; −21.5%) were observed through Week 53. Nineteen of 20 (95.0%) patients reported improved QoL.

**Conclusions:**

Velaglucerase alfa was well-tolerated in Chinese patients with GD1, with no new safety signals, and resulted in improved hemoglobin and platelet levels, as well as liver and spleen volumes.

## Highlights

• Limited data assess velaglucerase alfa in Chinese patients with Gaucher disease.
• Velaglucerase alfa over one year was well-tolerated with no new safety signals.
• Patient hemoglobin concentrations and platelet levels were increased from baseline.
• Patient liver and spleen volumes, enlarged at baseline, decreased over one year.
• Nearly all patients reported improved quality of life after one year of treatment.

## Graphical abstract

**Figure:**
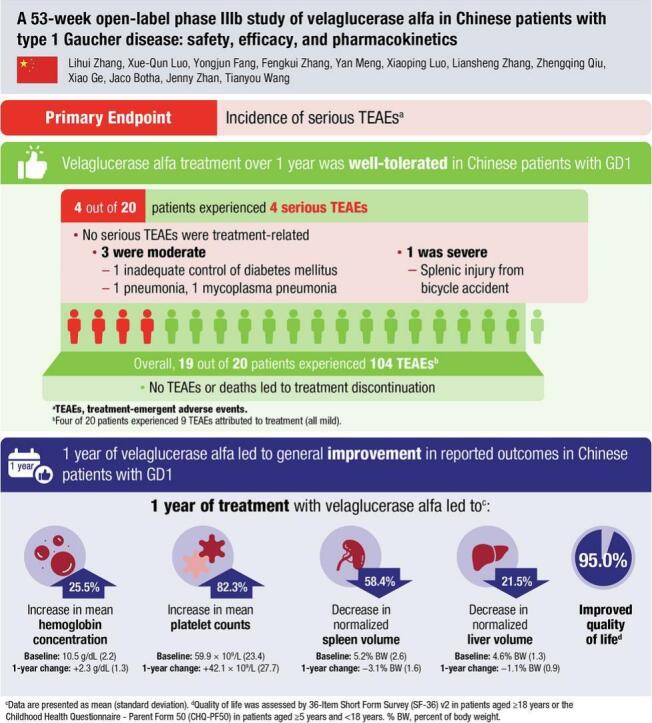

## 1 Introduction

Gaucher disease (GD) is a rare, hereditary autosomal recessive lysosomal disorder caused by pathogenic variants of the *GBA1* gene. *GBA1* variants cause deficiency of the β-glucocerebrosidase enzyme and subsequent glucocerebroside accumulation within the lysosomes of macrophages, transforming them into Gaucher cells [1], [2]. Gaucher cells are enlarged macrophages that primarily infiltrate liver, spleen, and bones, leading to enlargement and/or dysfunction of these organs [2], [3]. Clinical manifestations of GD include splenomegaly, hepatomegaly, anemia, thrombocytopenia, and deficiencies in coagulation factors [4]. Skeletal manifestations include bone mineral loss leading to osteopenia, osteoporosis, and osteonecrosis [4].

GD is typically subdivided into three types, with type 1 (GD1) being most common and having the least severe manifestations. GD1 presents with variable age of onset and without neurological manifestations [4]. Globally, the incidence of GD1 ranges from 0.45 to 22.9 cases per 100,000 live births and prevalence ranges from 0.26 to 0.63 per 100,000 persons [5]. In China, GD (of all types) is rare; limited data estimate the incidence ranges from 0.92 to 1.24 cases per 100,000 live births and the prevalence from 0.15 to 0.22 per 100,000 persons [6], [7], [8], [9]. In China, a limited number of hospitals can measure enzyme activity for lysosomal storage disorders such as GD and voluntary newborn screening occurs at a provincial level but is not mandated nation-wide [10], [11], [12], suggesting that GD is likely to be underdiagnosed.

Historically, patients received symptomatic treatment to alleviate multisystemic manifestations of GD. Current treatment aims to alleviate disease symptoms, prevent or relieve complications, and improve overall patient quality of life (QoL) [3]. A common approach is enzyme replacement therapy (ERT) that provides recombinant β-glucocerebrosidase targeted at macrophages, replacing deficient enzymatic activity and enabling degradation of glucocerebrosides. ERT is effective at improving most clinical manifestations of GD1 [3]. An alternative to ERT is substrate reduction therapy (SRT), which aims to reduce the synthesis of glucocerebroside [3]. Three ERTs have been approved by the US Food and Drug Administration, including velaglucerase alfa, which was approved in the United States and European Union in 2010 for long-term treatment of pediatric and adult patients with GD1 [3]. At the time of this study, one SRT and two ERTs, including velaglucerase alfa, were available for patients with GD in China [13], [14], [15]. Velaglucerase alfa was on the list of overseas new drugs in urgent clinical need in 2018 and was given approval in April 2021.

Currently, limited data exist on velaglucerase alfa treatment in Chinese patients with GD. In the absence of specific data on Chinese patients, insights may be drawn from studies conducted in other Asian populations. A post-marketing surveillance study in Japan assessed the safety and efficacy of velaglucerase alfa in Japanese patients with GD. Over a 6-year period, the study reported that velaglucerase alfa was well-tolerated by Japanese patients, and correlated with increased hemoglobin and platelet levels in addition to decreased liver and spleen volumes [16].

Several differences exist between Chinese and non-Chinese populations. One previous study suggests that GD1 often presents with a severe phenotype among Chinese patients, with early onset and severe hepatosplenomegaly and hematological complications [17]. However, given the phenotypic variability of GD1, ranging from presymptomatic patients to those with severe disease [4], [18], along with the limited availability of disease screening in China [12], it cannot be ruled out that Chinese patients who present with milder forms of GD may remain underdiagnosed. Nonetheless, Chinese patients were also found to have different pathogenic variants in the *GBA1* gene compared with those in Jewish and non-Jewish patients [17], [19]. The spectrum of *GBA1* variants in Chinese patients may have an impact on treatment outcomes in this population. Given the lack of clinical data as well as the diverse *GBA1* variants underlying GD1 in Chinese patients, the capacity for ERTs like velaglucerase alfa to improve patient outcomes in this population requires further evaluation.

## 2 Aim

As part of the approval requirements of China's Health Authority, this phase IIIb interventional post-approval study was conducted to evaluate the safety, efficacy, and pharmacokinetics (PK) of velaglucerase alfa in Chinese patients with GD1.

## 3 Methods

### 3.1 Study design

**Fig. 1:**
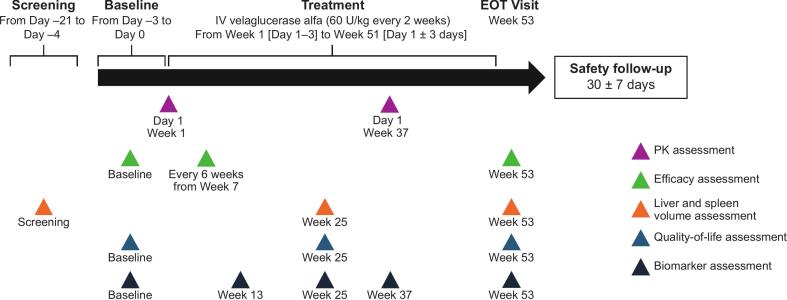
Study design. EOT, end of treatment; IV, intravenous; PK, pharmacokinetic.

This was a phase IIIb, multicenter, open-label, single-arm study of velaglucerase alfa in Chinese patients with a confirmed diagnosis of GD1 (ClinicalTrials.gov: NCT05529992; EudraCT Number: 2022–002323-35). The study was conducted in nine clinical sites in China, and the study design is shown in Fig. 1. Briefly, patients received intravenous (IV) infusions of velaglucerase alfa (60 U/kg body weight [BW] over 60 min) every 2 weeks (Q2W) during the treatment period (Week 1, Days 1–3) through Week 51 (Day 1 ± 3 days); maximum 26 infusion per patient). Patients had an end-of-treatment visit at Week 53 or 2 weeks (±7 days) after last infusion. All patients underwent a safety follow-up period of 30 days (±7 days) after last infusion.

### 3.2 Study population

#### 3.2.1 Inclusion criteria

All patients were aged ≥4 years with a diagnosis of GD1 and were divided into two subgroups: those who did not receive any treatment for GD within 12 months before enrollment (treatment-naïve patients) and those who had been treated with imiglucerase for GD within 12 months before screening but not within 14 days before screening (previously treated patients). In addition, patients had one of the following GD-related hematological abnormalities: hemoglobin concentrations of ≥1 g/dL and/or a platelet count of <90 × 10^9^/L below the lower limit of normal for their age and sex, as well as at least 1 of the following GD-related visceral abnormalities: moderate splenomegaly, assessed by palpation (2–3 cm below the left costal margin), or by abdominal radiology scan, with spleen volume >5 times normal; and/or hepatomegaly, assessed by palpation or by abdominal radiology scan, with liver volume >1 times normal.

#### 3.2.2 Exclusion criteria

Patients were excluded if they had type 2 or 3 GD; a splenectomy or an active, clinically significant spleen infarction within the 12 months prior to screening; received treatment with any investigational device or drug within 30 days prior to screening (or within 5 half-lives of that study drug, whichever was greater); were receiving red blood cell growth factor (e.g., erythropoietin), chronic systemic corticosteroids, or had been on such treatment within the 6 months prior to screening; presented with non–GD-related anemia (e.g., folic acid deficiency); were pregnant; or if they had experienced a severe (grade 3 or higher) infusion-related hypersensitivity reaction (anaphylactic or anaphylactoid reaction) to any ERT (approved or investigational).

### 3.3 Endpoints and assessments

The primary endpoint was the incidence of serious treatment-emergent adverse events (TEAEs). TEAEs were defined as any event emerging or manifesting at or after the initiation of the study medication or any existing event that worsened in either intensity or frequency following exposure to study medication. Serious TEAEs were coded using the most recent version of the Medical Dictionary for Regulatory Activities (MedDRA) and were defined as TEAEs that resulted in death, were life-threatening, required or prolonged inpatient hospitalization, resulted in persistent or significant disability or incapacity, or caused a congenital anomaly or birth defect. The severity of a TEAE was classified as mild (Grade 1), moderate (Grade 2), or severe (Grade 3 and above). Secondary endpoints included the incidence of TEAEs, infusion-related reactions, and rates of binding (BAbs) and neutralizing (NAbs) antibody formation. To assess anti-drug antibody formation, blood samples were collected during treatment visits prior to velaglucerase alfa infusion at baseline, Weeks 13, 25, 37, and at the end of treatment visit (Fig. 1). Samples were evaluated for the presence of anti-drug antibodies at designated laboratories.

Efficacy endpoints included the change from baseline to Week 53 in hemoglobin concentrations, platelet counts, spleen and liver volume, and quality of life (QoL). Spleen and liver volume were assessed by MRI or CT scan at local imaging centers. The imaging modality remained consistent for each patient over time during the study period. QoL was assessed using the 36-Item Short Form Survey (SF-36) version 2 in patients aged ≥18 years or the Childhood Health Questionnaire-Parent Form 50 (CHQ-PF50) in patients aged ≥5 and <18 years. The CHQ-PF50 was completed by the patient's parent or caregiver. QoL was not assessed in patients aged <5 years.

Serum samples were analyzed for the velaglucerase alfa concentration using a validated enzyme-linked immunosorbent assay (ELISA). PK assessments were conducted on Day 1 (Weeks 1 and 37) in treatment-naïve patients and included maximum serum concentration (C_max_), time to maximum serum concentration (T_max_), area under the concentration-time curve from time 0 to infinity (AUC_inf_), half-life (T_1/2_), clearance (CL), and the apparent steady-state volume of distribution (V_ss_).

Biomarker assessment included the percentage change from baseline to Week 53 in serum chemokine (CC motif) ligand 18 (CCL18) and glucosylsphingosine (Lyso-Gb1). Blood samples were collected during treatment visits before velaglucerase alfa infusion at baseline, Weeks 13, 25, 37, and at the end of treatment visit (Fig. 1). Samples were evaluated for CCL18 and Lyso-Gb1 at designated laboratories.

### 3.4 Statistical analysis

Given that GD is a rare disease, no formal sample size calculations were carried out and the sample size of 20 patients was based on feasibility.Serious AEs were coded using MedDRA version 27.0. Serious TEAEs were summarized overall and by System Organ Class and Preferred Term. Patients were only counted once per System Organ Class and once per Preferred Term. Multiple events of the same type were combined for each patient; the worst severity or outcome for each event type is presented. Event rates were calculated using the safety population (*n* *=* 20) as the denominator, irrespective of dropouts during follow-up. The safety population was identical to the intention-to-treat population (*n* *=* 20).

Efficacy, QoL, and biomarker analyses were calculated using the intention-to-treat population (*n* = 20). PK parameter estimates in the PK analysis set (*n* = 16; included treatment-naïve patients in the intention-to-treat population) were computed with Phoenix® WinNonlin® software version 8.3 using non-compartmental methods from individual serum-concentration time data and actual times and dosing, where appropriate.

Data from patients who were previously treated with imiglucerase ERT and treatment-naïve patients were analyzed separately, as well as pooled. Data were further analyzed separately (and pooled) for age groups <18 years and age ≥18 years and male versus female. All statistical analyses were performed using SAS v9.4.

## 4 Results

### 4.1 Patient population

The study was conducted from January 3, 2023 through August 5, 2024. Of the 20 patients enrolled, 16 were treatment-naïve and 4 were previously treated. In total, 19 (95.0%) patients completed the study; 1 treatment-naïve patient withdrew from the study due to “withdrawal by patient.” All 20 patients received ≥80% of the 26 protocol-required completed infusions, with a median of 26.0 infusions (range, 22.0–26.0). Two (10.0%) patients missed 1 infusion each. Baseline characteristics are shown in Table 1.

**Table 1:** Demographic and baseline characteristics.

Characteristic	<18 years old (*n* = 15)	≥18 years old (*n* = 5)	Previously treated (*n* = 4)	Treatment-naïve (*n* = 16)	Total (*n* = 20)
Sex, n (%) Female Male	8 (53.3) 7 (46.7)	5 (100.0) 0	2 (50.0) 2 (50.0)	11 (68.8) 5 (31.3)	13 (65.0) 7 (35.0)
Age, years, median (range)	9.0 (4.0–16.0)	31.0 (18.0–36.0)	7.5 (5.0–10.0)	13.0 (4.0–36.0)	11.5 (4.0–36.0)
Aged <18 years, n (%)	15 (100)	0	4 (100.0)	11 (68.8)	15 (75.0)
BMI, kg/m^2^, median (range)	16.6 (14.5–21.1)	19.5 (17.6–20.5)	16.1 (15.9–16.6)	17.5 (14.5–21.1)	17.1 (14.5–21.1)
Hemoglobin concentration, g/dL, mean (SD)	10.5 (1.8)	10.5 (3.1)	10.7 (1.3)	10.4 (2.4)	10.5 (2.2)
Platelet counts, × 10^9^/L, mean (SD)	61.6 (25.4)	54.0 (15.4)	73.5 (13.5)	56.1 (24.5)	59.9 (23.4)
Normalized spleen volume, mean % BW (SD)^b^	4.9 (1.7)	6.1 (4.5)	4.8 (1.8)	5.3 (2.8)	5.2 (2.6)
Normalized liver volume, mean % BW (SD)^a^	4.5 (0.9)	4.8 (2.3)	3.9 (0.6)	4.7 (1.4)	4.6 (1.3)

Abbreviations: BMI, body mass index; BW, body weight; MN, multiple of normal; SD, standard deviation.

a Normal volume is 2.5% BW. Overall mean (SD) MN for liver volume was 1.8 (0.5) times normal.

b Normal volume is 0.2% BW. Overall mean (SD) MN for spleen volume was 25.9 (12.9) times normal.

### 4.2 Safety results

Four patients had a total of 4 serious TEAEs that required or prolonged hospitalization, none of which were considered related to the study medication. One previously treated patient had mycoplasma pneumonia (moderate) and 3 treatment-naïve patients had 1 serious TEAE each of pneumonia (moderate), inadequate control of diabetes mellitus (moderate), and splenic injury (severe, due to fall from bicycle). Overall, 19 of 20 (95.0%) patients experienced 104 TEAEs, the majority of which were mild (*n* = 14 patients [70%], *n* = 95 events [91.3%]) or moderate (*n* = 4 patients [20%], *n* = 8 events [7.7%]), with only 1 severe TEAE reported (bicycle accident). The most commonly reported TEAEs were upper respiratory tract infection (*n* = 14 patients [70.0%], *n* = 27 events [26.0%]), cough (*n* = 5 patients [25.0%], *n* = 5 events [4.8%]), and pyrexia (*n* = 3 patients [15.0%], *n* = 4 events [3.8%]). Four (20.0%) patients experienced 9 mild TEAEs that were considered to be related to the study medication by the investigator (supraventricular extrasystoles [*n* = 3 events], abdominal pain [*n* = 2 events], dizziness [*n* = 2 events], atrioventricular block second degree [*n* = 1 event], and blood bilirubin increase [*n* = 1 event]). One patient reported 2 infusion-related reactions (dizziness) that were mild in severity and non-serious. No deaths and no TEAEs leading to discontinuation of study medication occurred. There were no significant differences in the profile of TEAEs in patients aged <18 years and ≥18 years.

A total of 3 (15.0%) treatment-naïve patients developed BAbs and 1 (5.0%) patient developed NAbs after velaglucerase alfa treatment. At baseline, 1 (5.0%) previously treated patient had pre-existing BAbs and NAbs, although no elevated BAb or NAb responses were observed in this patient after velaglucerase alfa treatment. In these patients with anti-drug antibodies, there were no infusion-related reactions and no negative impact on the efficacy parameters.

### 4.3 Efficacy results

**Fig. 2:**
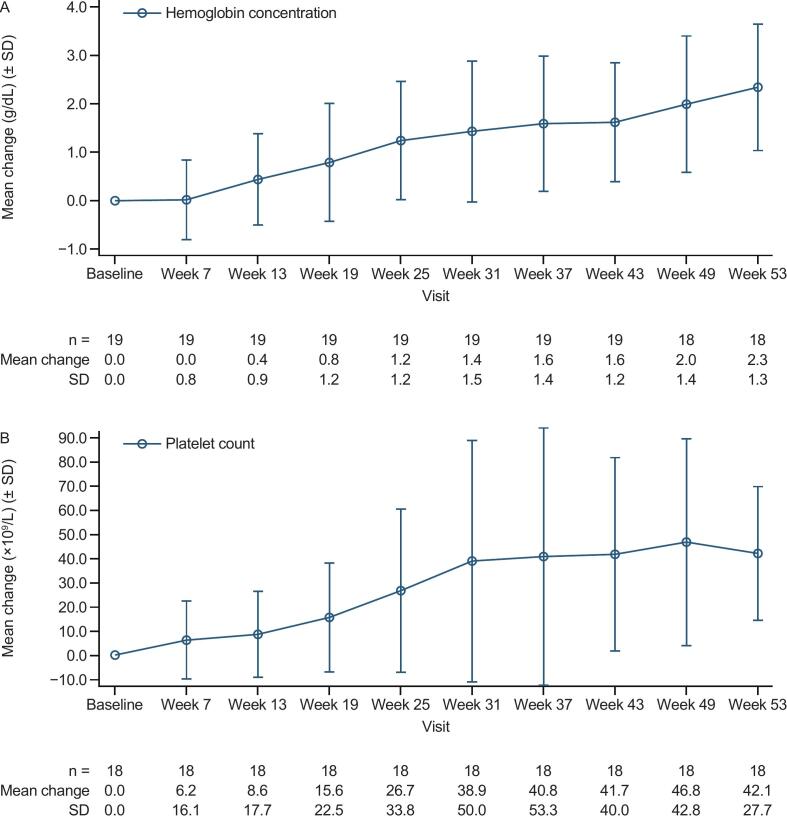
Mean (SD) changes from baseline in (A) hemoglobin concentrations and (B) platelet counts by study week in the ITT population. ITT, intention-to-treat; SD, standard deviation.

The mean percent changes in hemoglobin concentrations (*n* = 18) and platelet counts (*n* = 18) reflect improvements from baseline to Week 53, indicating a positive response to 1 year of treatment with velaglucerase alfa. Mean (standard deviation [SD]) hemoglobin concentrations increased from 10.5 g/dL (2.2) at baseline to 12.7 g/dL (2.0) at Week 53. The greatest mean (SD) increase in hemoglobin concentrations from baseline was observed at Week 53 (+2.3 g/dL [1.3], +25.5% [22.7]). Similarly, mean (SD) platelet counts increased from 59.9 × 10^9^/L (23.4 × 10^9^) at baseline to 106.5 × 10^9^/L (40.0 × 10^9^) at Week 53. The mean (SD) increase in platelet counts from baseline to Week 53 was 42.1 × 10^9^/L (27.7 × 10^9^/L), an increase of 82.3% (51.7). Mean changes from baseline in hemoglobin concentrations and platelet counts by study week are shown in Fig. 2.

**Fig. 3:**
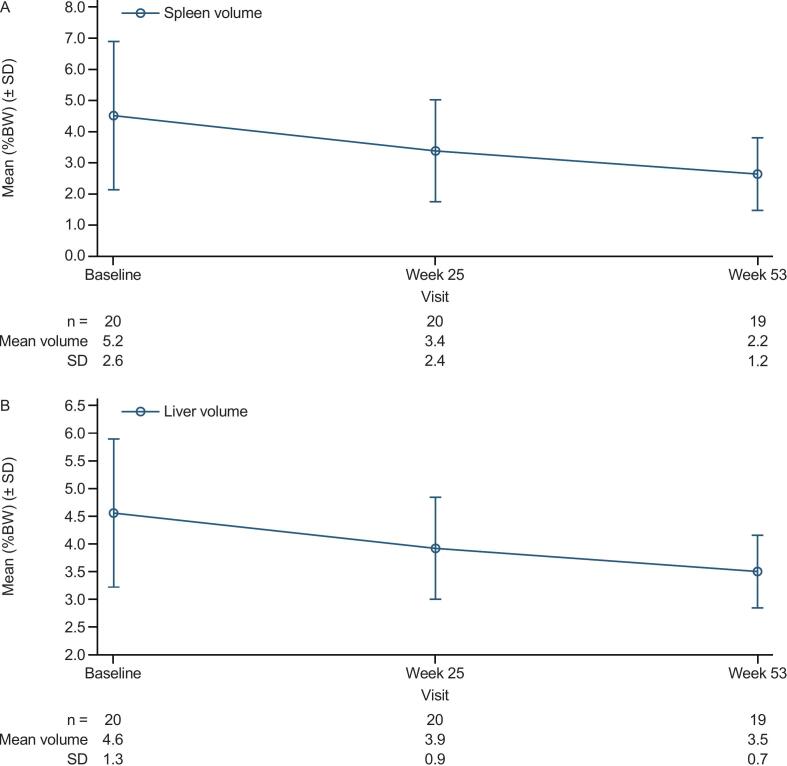
Mean (SD) normalized (A) spleen and (B) liver volume (% BW) over time. BW, body weight; SD, standard deviation.

Similarly, 1 year of treatment with velaglucerase alfa led to improved visceral outcomes in Chinese patients, with normalized spleen and liver volumes decreasing from baseline to Week 53 (*n* = 19) (Fig. 3). Mean (SD) normalized spleen volume decreased from 5.2% BW (2.6) at baseline to 2.2% BW (1.2) at Week 53 (normal volume, 0.2% BW). The greatest mean (SD) decrease in normalized spleen volume from baseline was observed at Week 53 (−3.1% BW [1.6], −58.4% [10.4]). Mean (SD) MN for spleen volume decreased from 25.9 (12.9) times normal at baseline to 10.7 (6.1) times normal at Week 53. Mean (SD) change and mean percent (SD) change in MN for spleen volume from baseline were −9.0 times normal (4.3) and −36.8% (12.7) at Week 25, and −15.4 (8.1) times normal and −58.4% (10.3) at Week 53. Mean (SD) normalized liver volume decreased from 4.6% BW (1.3) at baseline to 3.5% BW (0.7) at Week 53 (normal volume, 2.5% BW). The greatest mean (SD) decrease in normalized liver volume from baseline was observed at Week 53 (−1.1% BW [0.9], a decrease of 21.5% [13.5]). Mean (SD) multiples of normal (MN) for liver volume decreased from 1.8 (0.5) times normal at baseline to 1.4 (0.3) times normal at Week 53. Mean (SD) change and mean percent (SD) change in MN for liver volume from baseline were −0.3 times normal (0.3) and −12.6% (11.6) at Week 25, and −0.5 (0.4) times normal and −21.8% (13.5) at Week 53. Similar trends of efficacy parameters from baseline to Week 53 were observed in subgroups of previously treated and treatment-naïve patients, males and females, and in patients aged <18 years and ≥18 years (data not shown)*.*

Velaglucerase alfa-treated Chinese patients, or their parents or caregivers (for patients aged ≥5 and <18 years), filled out QoL surveys at the beginning and end of the study period. At baseline, 4/5 (80.0%) patients aged ≥18 years reported being “somewhat worse than 1 year ago,” whereas the majority of patients (11/15, 73.3%) aged <18 years reported their health being “about the same as 1 year ago” and 2 of 15 (13.3%) reported their health being “somewhat worse now than 1 year ago.” After 1 year of treatment with velaglucerase alfa, 3/5 (60.0%) patients aged ≥18 years reported being “somewhat better now than 1 year ago” and 1/5 (20.0%) reported being “much better now than 1 year ago.” QoL response at Week 53 was missing from 1 patient aged >18 years who withdrew from the study. Among patients aged <18 years, the majority (13/15, 86.7%) reported being “much better now than 1 year ago” and 2/15 (13.3%) patients reported being “somewhat better now than 1 year ago.” Overall, a majority of the study participants (19/20, 95.0%) reported improvement in QoL from baseline to Week 53.

### 4.4 PK results

**Fig. 4:**
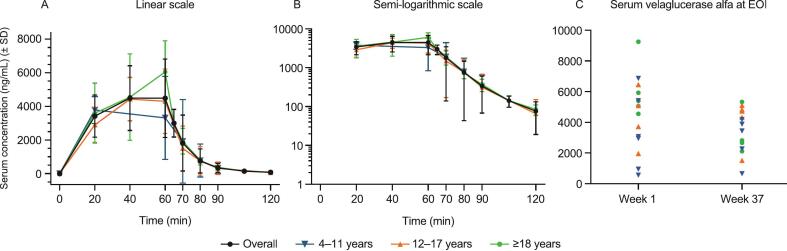
Mean (SD) concentrations of serum velaglucerase alfa over time displayed using (A) a linear scale or (B) a semi-logarithmic scale. (C) The concentration of serum velaglucerase alfa at EOI is shown for individuals at Weeks 1 and 37. EOI, end of infusion; SD, standard deviation.

The serum concentration of velaglucerase alfa was assessed and PK parameters were derived using a non-compartmental approach in treatment-naïve Chinese patients with GD1 after a 1-hour IV infusion. A total of 16 (80.0%) patients were included in the PK analysis set, including 5 patients aged ≥18 years, 5 patients aged 12–17 years, and 6 patients aged 4–11 years. Following a single IV dose of 60 U/kg velaglucerase alfa (Week 1), the mean serum velaglucerase alfa concentration-time profiles were generally comparable across age groups (4–11 years, 12–17 years, and ≥18 years), with C_max_ achieved by the end of infusion, followed by a rapid decrease (Fig. 4A,B). Furthermore, the concentrations of serum velaglucerase alfa at the end of the 60-min infusion were generally comparable between Week 1 and Week 37 (mean [SD]; Week 1, 4483.3 ng/mL [2249.2]; Week 37, 3451.7 ng/mL [1347.2]), suggesting that the PK of velaglucerase alfa were time-independent following Q2W dosing (Fig. 4C). Across age groups, mean CL ranged from 6.1 to 8.3 mL/min/kg, V_ss_ from 80.0 to 113.0 mL/kg, and T_1/2_ from 8.9 to 11.9 min (Table 2), similar to the previously reported PK profile of velaglucerase alfa in Caucasian patients [20].

**Table 2:** PK parameters (mean and %CV) for Week 1 by age group (PK analysis set).

Age group	T_max_^a^ (min)	C_max_ (ng/mL)	AUC_last_ (ng*min/mL)	AUC_inf_ (ng*min/mL)	T_1/2_ (min)	CL (mL/min/kg)	V_ss_ (mL/kg)
Overall, *n* = 16							
Mean (SD)	49.0 (19.0, 60.0)	5220.0 (1610.0)	233,000.0 (84,400.0)	235,000.0 (85,000.0)	10.1 (2.8)	7.3 (2.9)	96.3 (52.0)
%CV	34.0	30.7	36.3	36.1	27.8	39.5	54.0
≥18 years, *n* = 5							
Mean (SD)	58.0 (40.0, 59.0)	6140.0 (1870.0)	280,000.0 (110,000.0)	282,000.0 (110,000.0)	11.9 (1.5)	6.1 (2.5)	113 (78.7)
%CV	15.0	30.5	39.3	39.0	12.8	41.5	69.6
12–17 years, *n* = 5							
Mean (SD)	40.0 (39.0, 60.0)	4950.0 (1500.0)	223,000.0 (62,500.0)	224,000.0 (63,200.0)	8.9 (3.3)	7.3 (2.7)	99.2 (21.2)
%CV	20.7	30.4	28.1	28.2	37.1	37.0	21.4
4–11 years, *n* = 6							
Mean (SD)	39.5 (19.0, 60.0)	4690.0 (1360.0)	201,000.0 (70,500.0)	205,000.0 (73,400.0)	9.7 (2.9)	8.27 (3.4)	80.0 (46.9)
%CV	55.0	29.1	35.1	35.8	29.7	40.6	58.5

Abbreviations: AUC_inf_, area under the concentration-time curve from time 0 to infinity; AUC_last_, area under the concentration-time curve from time 0 to the final timepoint (120 min); CL, clearance; C_max_, maximum serum concentration; CV, coefficient of variation; PK, pharmacokinetics; T_1/2_, half-life; T_max_, time to maximum serum concentration; V_ss_, steady-state volume of distribution.

a Median (minimum, maximum).

### 4.5 Biomarker assessment results

**Fig. 5:**
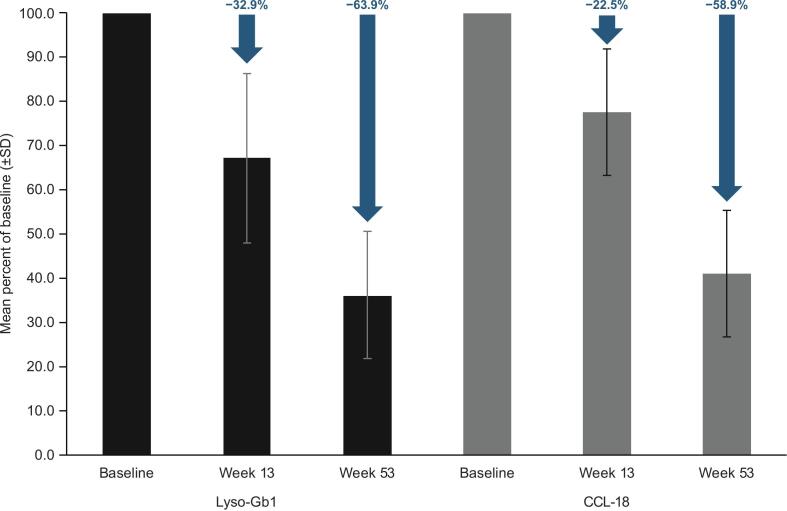
Mean (SD) percentage change from baseline in biomarkers. CCL18, serum chemokine (CC motif) ligand 18; Lyso-Gb1, glucosylsphingosine; SD, standard deviation.

During the study period, mean (SD) Lyso-Gb1 levels decreased from 306.5 μg/L (125.7) at baseline to 194.1 μg/L (72.7) at Week 13 and 103.8 μg/L (48.1) at Week 53, reflecting decrements in Lyso-Gb1 of 32.9% (19.2) and 63.9% (14.4), respectively. Similarly, mean (SD) CCL18 levels were reduced from 9.7 × 10^5^ ng/L (3.5 × 10^5^) at baseline to 7.4 × 10^5^ ng/L (2.6 × 10^5^) at Week 13 and 3.8 × 10^5^ ng/L (1.5 × 10^5^) at Week 53, reflecting reductions in CCL18 levels of 22.5% (14.3) and 58.9% (14.3). Collectively, the decreases in Lyso-Gb1 and CCL-18 levels seen at Week 13 were maintained through Week 53 (Fig. 5).

## 5 Discussion

Treatment with velaglucerase alfa Q2W over 1 year was well-tolerated in Chinese patients with GD1, with no new safety signals observed. There was a general increase in hemoglobin concentrations and platelet counts and a decrease in normalized liver and spleen volumes from baseline to Week 53, indicating a positive treatment response to velaglucerase alfa in Chinese patients with GD1. Mean serum velaglucerase alfa concentration-time profiles were generally comparable across age groups of 4–11 years, 12–17 years, and ≥18 years, in line with the known PK profile of velaglucerase alfa [20].

Our results are consistent with published literature in other patient populations. For example, a phase III study conducted in 5 centers in Argentina, Israel, Paraguay, the Russian Federation, and Tunisia reported a significant increase in hemoglobin concentration from baseline to 1 year in 12 treatment-naïve patients with GD1 (aged 4–62 years) who were treated with velaglucerase alfa 60 U/kg [21]. The study also reported a significant increase in platelet counts, significant decreases in mean spleen volume and CCL18 levels, a numerical decrease in liver volume, and found velaglucerase alfa (60 U/kg) to be generally well tolerated as a first-line treatment. A second open-label study (NCT00478647) assessed the safety and efficacy of 12 months of velaglucerase alfa treatment in 40 patients (aged 9–71 years) with GD1 who were previously treated with imiglucerase [22]. The results showed that switching from imiglucerase to velaglucerase alfa was generally well tolerated and hemoglobin concentrations, platelet counts, and spleen and liver volumes remained stable through 12 months.

In this study, both treatment-naïve and previously treated patients showed improvements in all clinical parameters. Alternatively, in the previous open-label study (NCT00478647), patients who switched from imiglucerase to velaglucerase alfa exhibited hemoglobin concentrations, platelet counts, and spleen and liver volumes that remained stable through 12 months but without notable improvement [22]. One likely explanation is that only 4 of 40 (10.0%) patients in the previous study received velaglucerase infusions ≥60 U/kg, whereas all patients in the current study were treated at the 60 U/kg dose [22]. A caveat is that only 4 patients with prior imiglucerase therapy were included in the current study, which constrains the extension of these findings to larger populations of previously treated patients with GD.

Alternatively, the incidence of anti-drug antibodies in our study was generally consistent with previous reports. In this study, 1 previously treated patient had pre-existing BAb and NAb, but no elevated BAb or NAb responses were observed after treatment. Furthermore, the presence of anti-drug antibodies in this study had no negative effect on clinical outcomes, nor did they lead to infusion-related reactions in any patient. This is similar to a previous report summarizing the effect of velaglucerase alfa in 289 patients with GD across 10 studies, including both treatment-naïve and patients who switched from imiglucerase, which found that anti-drug antibodies in patients exposed to velaglucerase alfa similarly had no observable effect on clinical or safety outcomes [23]. Together, the results of the aforementioned studies in adults and children across ethnicities support our results in both treatment-naïve Chinese patients and those who switched from imiglucerase therapy [21], [22], [23].

Several studies have reported the long-term safety and effectiveness of velaglucerase alfa. Specifically, two extension studies (NCT00391625, NCT00635427) have reported continued improvements over 2 years in patients with GD1, and over 5.8 years in treatment-naïve patients with GD1 [24], [25]. The longest follow-up to date is 12-year data from the Gaucher Outcome Survey [26]. Collectively, these studies suggested that the majority of adverse events related to velaglucerase alfa treatment are mild in nature, with serious adverse events attributed to velaglucerase alfa being observed in a minority (0.2%) of patients [24], [26]. In addition, post-marketing surveillance data (NCT03625882) from patients with GD types 1–3 in Japan indicated that long-term treatment (6 years) with velaglucerase alfa was well tolerated and associated with increased platelet counts, consistent with studies in patients outside of Asia [16]. Together, these results show that velaglucerase alfa offers a valuable long-term therapeutic option for both Chinese adults and children with GD1.

Furthermore, Lyso-Gb1, a deacylated metabolite of glucocerebroside, is a promising biomarker for diagnosis and treatment response in patients with GD [27], [28], [29]. In our study, levels of Lyso-Gb1 decreased early in treatment and were maintained through Week 53. This supports the use of plasma Lyso-Gb1 concentration as a useful biomarker for treatment response in Chinese patients with GD, although results may vary in different ethnic populations. Indeed, an observational cohort study in Japanese versus non-Japanese patients with GD showed that Lyso-Gb1 concentrations observed in Japanese patients were numerically lower than those observed in non-Japanese patients with GD receiving ERT [30].

Plasma CCL18 concentration is another biomarker that can be useful for diagnosing GD1 as well as treatment response. CCL18 is a chemokine involved in leukocyte chemotaxis that is prominently released from Gaucher cells and has been shown to correlate with splenic and liver volume as well as platelet counts in patients with GD1 [31], [32]. In this study, CCL18 levels decreased rapidly in Chinese patients after velaglucerase alfa induction, which persisted through Week 53, similar to general observations from prior studies [22], [33], [34]. However, unlike Lyso-Gb1, CCL18 release is not specific to GD [35], and therefore may be more useful as a surrogate marker.

All in all, improving our understanding of the pathogenic (or genetic) variants in Chinese patients with GD and the relationship between genotype and phenotype will be useful for incorporating biomarkers for disease prognosis of patients and guiding optimal therapeutic approaches [17], [36].

### 5.1 Strengths and limitations

This study has several strengths, including the comprehensive evaluation of safety, efficacy, and PK in treatment-naïve and previously treated patients. Other strengths include that the study had a population with a diverse age range from 4 to 36 years at the time of informed consent, and that treatment compliance was generally high, with all patients receiving ≥80% of the 26 protocol-required completed infusions. As well, this was the first study to demonstrate that the PK profile of velaglucerase alfa in Chinese patients is comparable with previously published data in Caucasian patients from clinical trials [20].

The study also has some limitations, including the small number of patients, that a genetic variant analysis was not conducted, and that quantitative chemical shift imaging, the most sensitive biomarker to assess bone marrow involvement in GD, was not included in this study, as widespread use is limited by test availability [37]. Additionally, the study was an open-label, single-arm design, and thus it was not possible to compare the results from velaglucerase alfa–treated patients against a placebo-treated control group. Also, due to the small number of patients available, only limited subgroup analyses between pediatric and adult patients were possible. Finally, either MRI or CT scans were used to assess organomegaly at local imaging centers, whichever was available, which prevented the consistent use of a single imaging modality across all patients. Nonetheless, the same imaging modality was used for each patient over the course of the study, which allowed reliable assessment of change in organ volume from baseline.

## 6 Conclusions

One year of treatment with velaglucerase alfa was well-tolerated in Chinese patients with GD1, with no new safety signals observed. The safety profile was consistent with either the clinical manifestations of GD or the known safety profile of velaglucerase alfa. In addition, 1 year of treatment with velaglucerase alfa resulted in a general increase in hemoglobin concentrations and platelet counts and a decrease in normalized liver and spleen volumes, suggesting that it is effective in these patients. Additionally, the GD1 biomarkers, Lyso-Gb1 and CCL18, decreased as early as Week 13 and were stable through Week 53. Finally, following a single IV dose of 60 U/kg velaglucerase alfa (Week 1), there was no clinical meaningful difference in exposure across age groups. The serum concentrations at the end of infusion were comparable between Week 1 and Week 37, suggesting that the PK properties of velaglucerase alfa were time-independent following Q2W dosing. The findings described in this report are consistent with those reported in previous studies among different patient populations.

## Data sharing statements

The datasets, including the redacted study protocol, redacted statistical analysis plan, and individual participant data supporting the results reported in this article, will be made available within 3 months from initial request, to researchers who provide a methodologically sound proposal. The data will be provided after their de-identification, in compliance with applicable privacy laws, data protection, and requirements for consent and anonymization.

## Research support

Study funding was received from Takeda Development Center Americas, Inc.

## Relationships

Xiao Ge, Jaco Botha, and Jenny Zhan are employees of Takeda and hold Takeda stocks and/or stock options.

## Patents and intellectual property

There are no patents to disclose.

## Other activities

There are no additional activities to disclose.

## CRediT authorship contribution statement

**Lihui Zhang:** Writing – review & editing, Writing – original draft, Investigation. **Xue-Qun Luo:** Writing – review & editing, Writing – original draft, Investigation. **Yongjun Fang:** Writing – review & editing, Writing – original draft, Investigation. **Fengkui Zhang:** Writing – review & editing, Writing – original draft, Investigation. **Yan Meng:** Writing – review & editing, Writing – original draft, Investigation. **Xiaoping Luo:** Writing – review & editing, Writing – original draft, Investigation. **Liansheng Zhang:** Writing – review & editing, Writing – original draft, Investigation. **Zhengqing Qiu:** Writing – review & editing, Writing – original draft, Investigation. **Xiao Ge:** Writing – review & editing, Writing – original draft, Conceptualization. **Jaco Botha:** Writing – review & editing, Writing – original draft, Formal analysis. **Jenny Zhan:** Writing – review & editing, Writing – original draft, Conceptualization. **Tianyou Wang:** Writing – review & editing, Writing – original draft, Investigation, Conceptualization.

## Patient consent statement

All patients or their guardians provided written informed consent prior to any study-related assessments and procedures.

## Ethics statement

This study was conducted in compliance with Good Clinical Practice regulations and guidelines, and all applicable local regulations. The clinical study protocol, protocol amendment, the investigator's brochure, a sample informed consent form, and other study-related documents were reviewed and approved by the ethics committees at each study site.

## Funding

Study funding was received from Takeda Development Center Americas, Inc. and the funding to the investigators was administered via the institution administration office, per local practice.

## Declaration of competing interest

Xiao Ge, Jaco Botha, and Jenny Zhan are employees of Takeda and hold Takeda stocks and/or stock options. The other authors declare no conflicts of interest. Study funding was received from Takeda Development Center Americas, Inc.

## Data Availability

Data will be made available on request.
